# Unraveling the antimicrobial potential of *Lactiplantibacillus plantarum* strains TE0907 and TE1809 sourced from *Bufo gargarizans*: advancing the frontier of probiotic-based therapeutics

**DOI:** 10.3389/fmicb.2024.1347830

**Published:** 2024-02-14

**Authors:** Feiyun Huang, Yanni Zhao, Yusen Hou, Yu Yang, Bisong Yue, Xiuyue Zhang

**Affiliations:** ^1^Key Laboratory of Bio-Resources and Eco-Environment (Ministry of Education), College of Life Sciences, Sichuan University, Chengdu, China; ^2^School of Pharmacy, Chengdu University of Traditional Chinese Medicine, Chengdu, China; ^3^Sichuan Key Laboratory of Conservation Biology on Endangered Wildlife, College of Life Sciences, Sichuan University, Chengdu, China

**Keywords:** *Bufo gargarizans*, *Lactiplantibacillus plantarum*, SCFAs, bacteriocin, GC-MS, whole-genome sequencing

## Abstract

**Introduction:**

In an era increasingly defined by the challenge of antibiotic resistance, this study offers groundbreaking insights into the antibacterial properties of two distinct *Lactiplantibacillus plantarum* strains, TE0907 and TE1809, hailing from the unique ecosystem of *Bufo gargarizans*. It uniquely focuses on elucidating the intricate components and mechanisms that empower these strains with their notable antibacterial capabilities.

**Methods:**

The research employs a multi-omics approach, including agar diffusion tests to assess antibacterial efficacy and adhesion assays with HT-29 cells to understand the preliminary mechanisms. Additionally, gas chromatography-mass spectrometry (GC-MS) is employed to analyze the production of organic acids, notably acetic acid, and whole-genome sequencing is utilized to identify genes linked to the biosynthesis of antibiotics and bacteriocin-coding domains.

**Results:**

The comparative analysis highlighted the exceptional antibacterial efficacy of strains TE0907 and TE1809, with mean inhibitory zones measured at 14.97 and 15.98 mm, respectively. A pivotal discovery was the significant synthesis of acetic acid in both strains, demonstrated by a robust correlation coefficient (cor ≥ 0.943), linking its abundance to their antimicrobial efficiency. Genomic exploration uncovered a diverse range of elements involved in the biosynthesis of antibiotics similar to tetracycline and vancomycin and potential regions encoding bacteriocins, including Enterolysin and Plantaricin.

**Conclusion:**

This research illuminates the remarkable antibacterial efficacy and mechanisms intrinsic to *L. plantarum* strains TE0907 and TE1809, sourced from *B. gargarizans*. The findings underscore the strains' extensive biochemical and enzymatic armamentarium, offering valuable insights into their role in antagonizing enteric pathogens. These results lay down a comprehensive analytical foundation for the potential clinical deployment of these strains in safeguarding animal gut health, thereby enriching our understanding of the role of probiotic bacteria in the realm of antimicrobial interventions.

## 1 Introduction

Gastrointestinal (GI) disorders, predominantly triggered by bacterial pathogens like *Pseudomonas aeruginosa, Campylobacter jejuni, Escherichia coli*, critically impact global health, with their management complicated by escalating antibiotic resistance worldwide, and it is urgent to develop new green antibiotic products (Greenwood-Van Meerveld et al., 2017; Zhao et al., 2017; Yang et al., 2022). Probiotic lactic acid bacteria (LAB) emerge as potential antibiotic alternatives, effectively inhibiting enteric pathogens for both prevention and treatment. Nonetheless, given the complex and varied composition of gastrointestinal microbiota in humans and animals, coupled with the diversity of probiotic strains and their individualized effects, the study of the interaction mechanisms between probiotics and other microbial communities is still in its nascent phase (Gomaa, 2020). Therefore, there is a critical need for comprehensive research to uncover the inhibitory constituents of probiotics, aimed at facilitating strategic and efficacious colonization of beneficial elements in human and animal gastrointestinal tracts, crucial for maintaining gut microbiota balance and suppressing the growth of intestinal pathogens.

*Lactobacillus*, a genus within the lactic acid bacteria (LAB) family, is a cornerstone of probiotic research, acclaimed for its vital role in fermentation and significant health benefits, such as enhancing gut flora and boosting immunity (Ballini et al., 2023). With over 220 species, it is the most expansive and diverse LAB group (http://www.bacterio.net/lactobacillus.html). Research shows *Lactobacillus* species exhibit varied genetic and physiological traits across different environments. Some, like *Lactobacillus delbrueckii* and *Lacticaseibacillus rhamnosus*, occupy specialized niches, while *Lactiplantibacillus plantarum* is remarkably versatile, thriving in diverse habitats from dairy to human tracts, reflecting its ability to adapt metabolically (Valan Arasu et al., 2015; Berbegal et al., 2016; Min Hsiu et al., 2016; Mendoza et al., 2023; Rebaza-Cardenas et al., 2023). Moreover, Endorsed by the European Food Safety Authority (EFSA) and the U.S. Food and Drug Administration (FDA) for its safety, *L. plantarum* is a versatile probiotic, widely employed in fermenting diverse foods and drinks, and in medical and pharmaceutical applications (Todorov and Franco, 2010; Capozzi et al., 2012). Its health-promoting and antibacterial qualities have not only led to innovative probiotic products but also enhanced food safety through biopreservation (Tang et al., 2023).

Numerous studies have focused on investigating the potential impact of *L. plantarum* in managing diverse gastrointestinal disorders, including infectious diarrhea, antibiotic-associated diarrhea, irritable bowel syndrome (IBS), and inflammatory bowel disease (Ji et al., 2023; Kumar et al., 2023; Yang et al., 2023). *L. plantarum* 299v ameliorated *Escherichia coli*-induced intestinal permeability in rats, mitigating a 53% surge in mannitol permeation through the intestinal barrier, as gauged using Ussing chambers (Mangell et al., 2002). Similarly, Yang et al. (2014) revealed that *L. plantarum* CGMCC 1258 effectively combats enterotoxigenic *E. coli*-induced diarrhea in young pigs by bolstering intestinal integrity and regulating tight junction proteins (Yang et al., 2014). Nevertheless, the current body of research on the probiotic mechanism of *L. plantarum* still needs to be improved due to existing technical limitations. Despite its notable colonization capabilities and ability to effectively target various pathogens, further investigation is required to comprehend its potential fully.

Currently, the integration of multi-omics sequencing methodologies, proficiently addresses the constraints inherent to *in vitro* assays of LAB, enabling a profound elucidation of its antimicrobial mechanisms and salutary impacts. The availability of complete genome sequences derived from *L. plantarum* DNA strains permitted us to understand better this LAB species' potential for adaptation and functionality. The complete genome sequencing of *L. plantarum* strains, starting with the human saliva strain *L. plantarum* WCFS1, has significantly deepened insights into LAB's adaptability and functionality (Kleerebezem et al., 2003). This genomic revelation, followed by a series of sequenced genomes, has facilitated a comprehensive understanding of LAB's evolutionary journey and environmental interactions through comparative genomics. Siezen et al.'s (2010) study elegantly dissected the genomic landscape of *L. plantarum* across 42 strains, shedding light on its exceptional adaptability. They identified a core set of 2,049 genes common to all strains, with 121 unique genes distinguishing *L. plantarum* from other LAB species. Comparative analysis revealed gene variations compared to the WCFS1 genome, with many distinctive genes located in lifestyle adaptation regions, highlighting the bacterium's evolutionary ingenuity (Siezen et al., 2010). Furthermore, in metabolomics, the integration of gas chromatography, gas chromatography-mass spectrometry, and high-performance liquid chromatography is paramount for the precise quantification of short-chain fatty acids, owing to their exceptional volatility and versatility, finding extensive utility in medical and chemical research domains (De Preter et al., 2009; De Baere et al., 2013; Mulat and Feilberg, 2015; Marques et al., 2023).

Residing in the Chordata phylum and Bufonidae family, *Bufo gargarizans*, a medicinal amphibian indigenous to China, has garnered significant academic interest for its anti-inflammatory and anti-neoplastic properties, particularly in its secretions and dermal extracts (Wang et al., 2012; Qi et al., 2014). This species, characterized by its rich integumentary and digestive microbiota, stands at the forefront of probiotic-related research. While literature on its probiotic associations remains sparse, studies on amphibian-derived probiotics offer valuable perspectives (Kueneman et al., 2016; Antwis and Harrison, 2018).

Our research team, having sourced specimens of *B. gargarizans* from the Jiuzhaigou Valley Scenic and Historic Interest Area in Sichuan Province, pioneered the isolation and screening of two prominent *L. plantarum* strains from their intestinal microbiota. In transcending traditional phenotypic analyses of LAB strains, this study meticulous control experiments, genomic profiling, and metabolomic explorations collectively decode the antibacterial dynamics inherent to *L. plantarum* originating. Notably, our novel undertakings in genomic visualizations and assessments of short-chain fatty acid synthesis chart unprecedented territory in this field. Collectively, our findings constitute a robust multi-omics testament to the clinical significance of these *Lactobacillus* strains in elucidating molecular mechanisms and bolstering intestinal fortification.

## 2 Materials and methods

### 2.1 Experimental methods

#### 2.1.1 Experimental strains

We selected the *L. plantarum* strains TE0907 and TE1809 (Supplementary material S1), stemming from preliminary *in vitro* screenings within our facility, originally derived from the intestinal milieu of mature *B. gargarizans* specimens. These initial evaluations vouched for their commendable safety profile, manifesting through the absence of hemolysis, susceptibility to a septet of prevalent antibiotics (Clindamycin, Clarithromycin, Gentamicin, Chloromycetin, Tetracycline, Ampicillin, Ceftriaxone) (Supplementary material S2), and marked gastrointestinal resilience. Currently, these strains have been meticulously conserved at the esteemed China General Microbiological Culture Collection Center (CGMCC), with their corresponding study sequences diligently archived in the Sequence Read Archive (SRA) of the National Center for Biotechnology Information (NCBI) website (https://www.ncbi.nlm.nih.gov/), accessible under the BioProject designation PRJNA1066612. The reference strain for the experiment was *L. rhamnosus* GG ATCC 53103 (LGG). This strain was graciously provided by Yijia Biotechnology Co. Ltd., Chengdu.

#### 2.1.2 Determination of bacterial strains' cell adhesion

HT-29 cells, post-subculturing, were standardized to 5 × 10^6^ cells/mL. In each culture plate well, 1 mL of this cell suspension was seeded and incubated at 37°C in a 5% CO_2_ environment until monolayer formation. Following DMEM medium removal and a PBS (Aoke, Chengdu) wash, 1 mL bacterial suspension was inoculated into each well in triplicate for every sample. After 2 h at the same conditions, residual bacterial suspensions were discarded. Wells were then thoroughly rinsed with PBS to eliminate unbound bacteria. Cells were detached utilizing 0.7 mL of 0.25% trypsin-EDTA (Aoke, Chengdu) for 10 min, halted by adding 0.3 mL DMEM. Subsequent cell suspensions were quantified using a 10-fold dilution method. Adhesion efficiency was assessed by comparing bacterial concentrations pre- and post-incubation.

#### 2.1.3 Pathogen susceptibility assay

The Oxford cup assay evaluated the antagonistic potential against various pathogens. Bacterial strains of interest, namely *E. coli* CMCC (B) 44102, *S. aureus* CMCC (B) 26003, *P. aeruginosa* CMCC (B), *C. jejuni* ATCC 33291, *C. coli* ATCC 43478, *S. typhimurium* ATCC 14028, *S. haemolyticus* ATCC29970, *Y. enterocolitica* ATCC 23715, *P. multocida* ATCC51689, *L. monocytogenes* ATCC 19115, and *Salmonella* paratyphi type B CMCC (B) 50094, were propagated in LB medium (Huankai, Guangdong) until the exponential phase. Post-cultivation, these strains were collected, rinsed with PBS, and adjusted to a 10^8^ CFU/mL final concentration. Petri dishes were prepared with 20 mL of LB agar (Huankai, Guangdong) enriched with 1.5% agarose for the assay. Upon solidification, three symmetrically spaced Oxford cups were anchored onto each plate, dispensing 200 μL of TE0907 and TE1809′s HMV18 cell-free supernatant (CFS). *L. rhamnosus* GG ATCC 53103 (LGG) was employed as a reference strain for comparative control. The inhibitory zone diameters of each strain were standardized using the *Z*-Score normalization technique.

#### 2.1.4 Exploration of antibacterial active ingredients

In order to further understand the effective ingredients involved in the excellent antibacterial properties of the two probiotics mentioned above, the conditioned culture supernatant (CFS) of the probiotics was prepared again. Utilizing a cohort of five meticulously selected animal pathogens as indicative models, the prepared cell-free supernatant (CFS) was subjected to a series of distinct, non-sequential interventions designed to elucidate the spectrum of antibacterial components. These interventions comprised the assay of hydrogen peroxide via specialized test strips (Huankai, Guangdong), thermal treatment in a 70°C precisely controlled water bath (Yiheng, Shanghai), pH normalization to 7 using a 1.0 M NaOH solution (Jinbohua, Tianjin), and the incorporation of proteinase K (Suolaibao, Beijing) into the CFS. This strategic approach was employed to dissect the antibacterial constituent profile inherent within these strains.

#### 2.1.5 GC-MS analysis of acid production capacity

We utilized a Shimadzu GCMS-QP2010 Plus gas chromatograph-mass spectrometer with a RESTEK Rtx-5 column (30 m × 0.25 mm × 0.25 μm). The temperature program started at 40°C for 5 min, increased to 150°C at 5°C/min, then to 280°C at 10°C/min, holding for 2 min. High-purity helium (>99.999%) was the carrier gas at 1.0 mL/min. Mass spectrometry parameters included electron impact ionization at 200°C, an interface temperature of 220°C, and a m/z 33–500 scan range. For sample preparation, fermentation broth (4 mL) was mixed with 10 μL of a 2-ethylbutyric acid internal standard (200 μg/mL). Samples were injected in split mode (1:3 ratio, 1 μL volume) at 270 μL with a 0.1-min solvent delay. This method quantified acids such as acetic, butyric, and valeric.

Furthermore, Pearson correlation analysis was employed to investigate the association between the concentrations of various short-chain fatty acids in separate bacterial strains and their average inhibitory effects against 11 tested pathogenic bacteria.

#### 2.1.6 Genome sequencing, genome annotation and gene annotation related to bacteriostasis

The genomes of *L. plantarum* TE0907 and *L. plantarum* TE1809 were sequenced using the HiSeqTM 2000 platform by Biotechnology Corporation in Shanghai, China. To ensure precise annotation information for the predicted protein-encoding genes, multiple databases, such as NR (Altschul et al., 1990), Swiss-Prot, GO, KEGG, and COG (Tatusov et al., 2003), were employed for Blast alignment. Furthermore, Circos (Krzywinski et al., 2009) generated a circular genome chromosome map that visualizes chromosome sequences, gene positions, mutation information, and other relevant data. Lastly, the BAGEL4 database (van Heel et al., 2018) was employed for predictive alignment of bacteriocin-related genes within the genome of LAB.

#### 2.1.7 Statistical analysis

Statistical analyses were performed using GraphPad Prism version 8 (California, USA), presenting data as means ± standard deviation (SD) and employing one-way ANOVA with the Student's *t*-test for comparisons. Significance was established at *P* < 0.05; all experiments were independently conducted and iterated at least thrice.

## 3 Results

### 3.1 Evaluation of adhesion rate of intestinal epidermis

**Figure 1:**
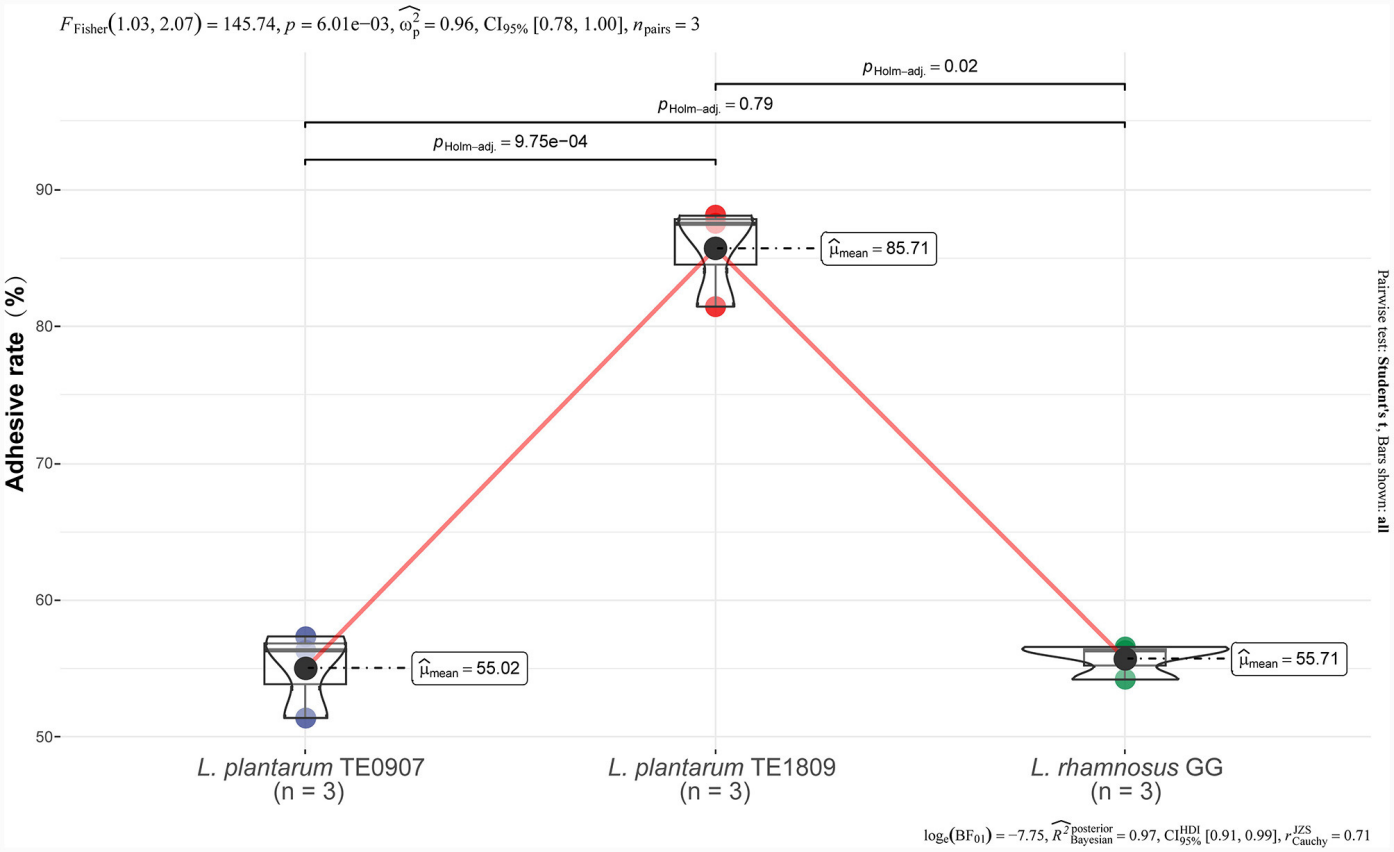
Adhesion rates of LGG, TE0907, and TE1809 to HT-29 cells.

TE0907 (55.02 ± 3.18%) exhibits no significant disparity when contrasted with LGG (55.71 ± 41.29%) (*P* > 0.05), while TE1809 (85.71 ± 3.70%) significantly surpasses LGG (Figure 1). Hence, at this preliminary stage, it can be inferred that the mentioned strains possess notable adhesion capabilities, implying their potential to colonize the intestinal epithelium, forming the basis for their prospective antibacterial functions.

### 3.2 Antibacterial activity assessment

**Figure 2:**
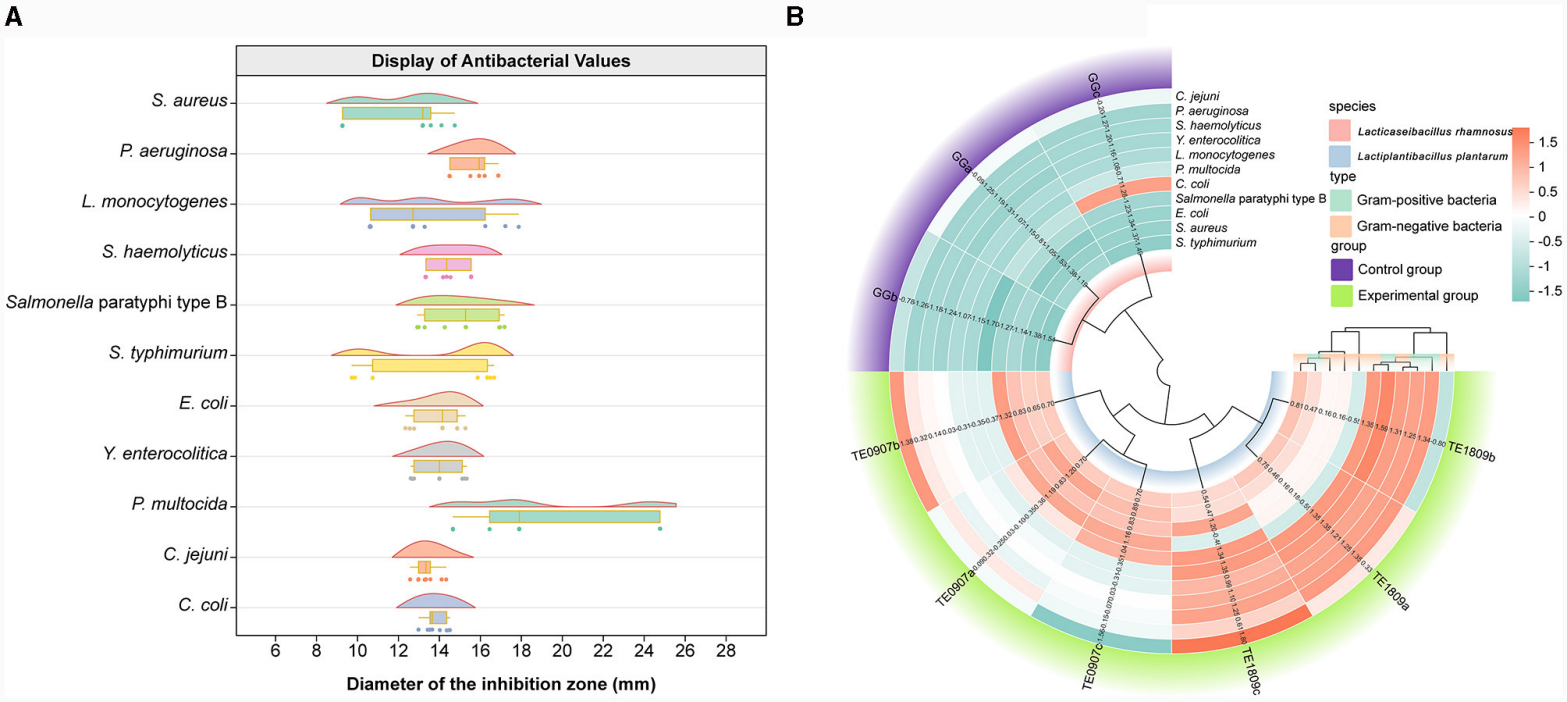
Antibacterial activity assessment. **(A)** LGG and the inhibitory diameter distribution chart of *L. plantarum* TE0907 and *L. plantarum* TE1809; **(B)** statistical diagram for bacteriostatic test of LGG and *L. plantarum* TE0907 and *L. plantarum* TE1809.

Prominent disparities were observed in the inhibitory prowess displayed by distinct LAB strains. In conjunction with the LGG strain, the two candidate strains manifested antagonistic capacities against pathogenic bacteria. Notably, a preponderance of these probiotics unveiled heightened antimicrobial efficacy against *P. multocida*, yet presented a subdued inhibitory response to *S. aureus* (Figure 2A). Contrastingly, TE0907(average value: 14.97 mm) and TE1809 (15.98 mm) outstripped LGG (12.56 mm) in their antimicrobial potency against an eclectic assortment of animal pathogens, especially underscoring their efficacy against gram-positive bacteria, thereby elucidating their broad-spectrum inhibitory attributes (Figure 2B). Intriguingly, our two strains of *L. plantarum*, when juxtaposed with the *L. plantarum* DY1 studied by Mao et al. (2020) (*E. coli*: 12.89 ± 0.21 mm; *Salmonella*: 15.93 ± 0.22 mm; *S. aureus*: 15.70 ± 0.41 mm), exhibited an equally compelling or even superior antibacterial zone diameter using a comparable method for antimicrobial performance testing. This denotes an unequivocal competitive edge in antimicrobial efficacy within the same species.

### 3.3 Exploration of antibacterial active ingredients

CFS showed no evidence of hydrogen peroxide production after evaluating hydrogen peroxide test strips. However, the antibacterial potency of CFS from both TE0907 and TE1809 was lost after pH adjustment, underscoring the primary role of organic acids in its antibacterial effect. Post proteinase K treatment, there was a marginal decline in the antibacterial activity of the strains, suggesting that *L. plantarum* might produce antibacterial peptides or proteins that play a minor role in their inhibitory action. Notably, following heat exposure to the CFS, the antibacterial efficacy of both strains remained largely intact against most tested bacteria (Table 1).

**Table 1:** Statistics of active antibacterial ingredients (a: *P* < 0.05; b: *P* < 0.01; c: *P* < 0.001; d: *P* < 0.0001).

**Strains**	**Treatment**	***S. aureus* (mm)**	***P. aeruginosa* (mm)**	***S. haemolyticus* (mm)**	***Salmonella* paratyphi type B (mm)**	***E. coli* (mm)**
*L. rhamnosus* GG	Blank	0.00	0.00	0.00	0.00	0.00
	CFS	9.20 ± 0.04	14.42 ± 0.20	12.67 ± 0.09	13.45 ± 0.70	13.63 ± 0.77
	pH 7.0	0.00d	0.00d	0.00d	0.00d	0.00d
	70°C	9.38 ± 0.13	13.10 ± 0.02d	12.25 ± 0.36	12.27 ± 0.22a	12.17 ± 0.23a
	Proteinase K	8.22 ± 0.01d	11.42 ± 0.01d	10.38 ± 0.05d	11.33 ± 0.21b	10.36 ± 0.06c
*L. plantarum* TE0907	Blank	0.00	0.00	0.00	0.00	0.00
	CFS	13.75 ± 0.41	16.18 ± 0.39	15.23 ± 0.21	16.27 ± 1.16	13.92 ± 0.23
	pH 7.0	0.00d	0.00d	0.00d	0.00d	0.00d
	70°C	13.37 ± 0.25	14.56 ± 0.04c	13.94 ± 0.4b	15.94 ± 0.17	13.21 ± 0.01
	Proteinase K	11.21 ± 0.03d	12.55 ± 0.27d	12.33 ± 0.28d	14.09 ± 0.05a	10.85 ± 0.37c
*L. plantarum* TE1809	Blank	0.00	0.00	0.00	0.00	0.00
	CFS	13.36 ± 0.28	17.06 ± 0.18	13.80 ± 0.95	14.29 ± 0.11	13.99 ± 0.77
	pH 7.0	0.00d	0.00d	0.00d	0.00d	0.00d
	70°C	13.51 ± 0.38	15.75 ± 0.07d	13.73 ± 0.16	14.37 ± 0.52	13.99 ± 0.12
	Proteinase K	9.26 ± 0.16d	12.42 ± 0.12d	12.39 ± 0.23a	9.78 ± 0.79c	12.32 ± 0.1b

### 3.4 Acid production capacity and acid production component analysis

**Figure 3:**
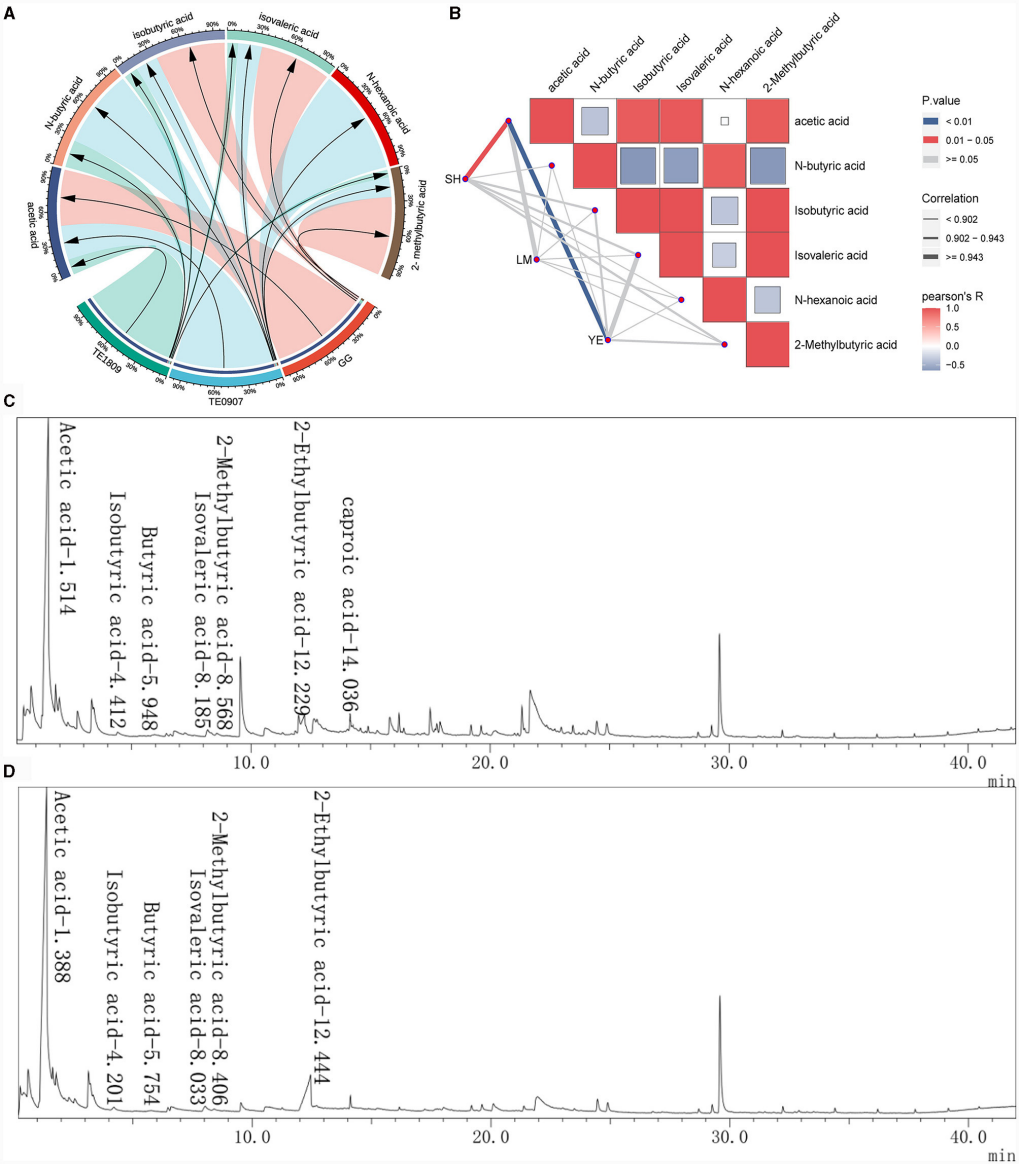
Study on the production of SCFAs by LGG and 2 LAB strains using GC-MS. **(A)** SCFAs content of each strain; **(B)** The correlation between the short-chain fatty acid content of each strain and the inhibitory effects on certain pathogens was analyzed using the R package ggcor (SH, *S. haemolyticus*; LM, *L. monocytogenes*; YE, *Y. enterocolitica*); **(C, D)** Total ion flow chromatogram for the determination of SCFAs in *L. plantarum* TE0907 and *L. plantarum* TE1809 using GC-MS.

GC-MS analysis identified acetic acid (3.576–13.08 μg/mL), N-butyric acid (0.018–0.052 μg/mL), isobutyric acid (0.06–0.18 μg/mL), isovaleric acid (0.061–0.41 μg/mL), and isocaproic acid 2-Methylbutyric acid (0.022–0.23 μg/mL) as the salient SCFAs, a revelation corroborated by Figure 3A total ion chromatogram, which adeptly profiles these SCFAs with commendable separation integrity, negligible tailing, and a harmonized baseline. The juxtaposition of the internal standard, 2-methylbutyric acid, with other pivotal peaks underscores the assay's unparalleled separation prowess, buttressing its robustness, especially in isolating SCFAs, a claim bolstered by the conspicuous absence of peak interference (Figures 3C, D). It is also paramount to note that acetic acid dominates the SCFAs landscape across all strains and exhibits a marked affinity with the levels of other acids. Furthermore, our study uncovered a robust correlation (correlation coefficient ≥ 0.943) between the antimicrobial effectiveness and the presence of acetic acid, a connection far more distinct compared to other short-chain fatty acids (SCFAs), which was significantly relevant in inhibiting *S. haemolyticus* and *Y. enterocolitica* (*P* < 0.05) (Figure 3B). Strains proficient in metabolizing robust concentrations of acetic acid may engender a pronouncedly acidic milieu, detrimental to the proliferation of pathogenic organisms, thereby effectuating a competitive exclusion dynamic.

### 3.5 Genomic analysis

After second-generation Illumina HiSeq sequencing and quality control, over 85% of the bases in the genomes of two bacterial strains were of high quality, ensuring reliable subsequent analysis. TE0907′s genome spans 3,154,032 base pairs with a 44.69% GC content and 3,055 protein-coding genes, while TE1809′s genome measures 3,294,124 base pairs, a 44.32% GC content and houses 3,227 protein-coding genes. The sequencing data is diligently archived in the Sequence Read Archive (SRA) of the National Center for Biotechnology Information (NCBI) website (https://www.ncbi.nlm.nih.gov/), and is accessible under the BioProject designation PRJNA1066612.

**Figure 4:**
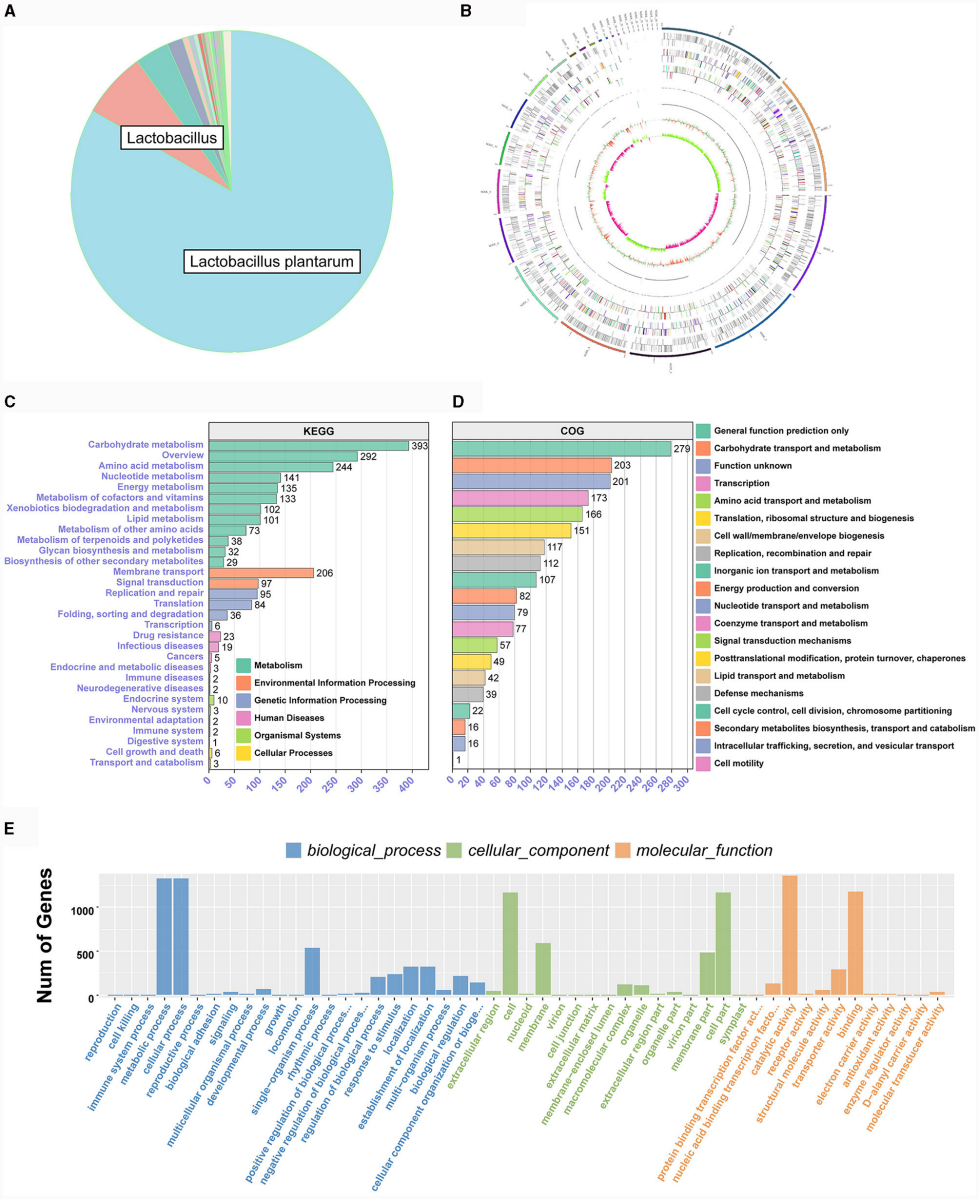
Genome functional annotation of *L. plantarum* TE0907. **(A)** Chromosome Circos plot of the *L. plantarum* TE0907 genome [annotations for the positive and negative strands are represented on the circular diagram's outer and inner sides, respectively. The outermost layer marks chromosomal DNA base positions. Layers two and three show protein-coding genes, while layers four to seven represent COG and KEGG functional annotations. Layers eight and nine cover GO annotations, and layers ten and eleven contain rRNA (5S, 16S, 23S), tRNA, and sRNA gene information. The twelfth layer displays relative GC content, and the thirteenth indicates GC skew; Color annotations are provided in Supplementary material S3; **(B–E)** NR, COG, GO, KEGG database annotation of the *L. plantarum* TE0907 genome based on NCBI Blast + alignment.

Both LAB strains exhibited extensive functional gene annotations, with 99.79% (2,910 genes) and 99.55% (3,067 genes) aligning to the NR database, predominantly corresponding to the *L. plantarum* species, consistent with prior 16S rRNA gene-based identifications (Figure 4A; Supplementary Figure S1A). We assembled the genomes of two bacterial strains and constructed their chromosomal circos plots (Figure 4B; Supplementary Figures S1B). The GO database identified 2,108 and 2,125 genes in these strains, mainly in the molecular function domain, highlighting their advanced biosynthetic aptitude. KEGG annotations revealed their affinity to metabolic pathways, with TE0907 and TE1809 having specific gene counts for glucose and amino acid metabolism and a notable focus on secondary metabolite production. COG analysis emphasized their protein synthesis prowess, with over 150 genes related to translation and ribosomal activities (Figures 4C–E; Supplementary Figures S1C–E). Additionally, the annotation outcomes of bacteriocin and immune-related genes in the KEGG database revealed that the genomes of the two strains exhibited the presence of genes associated with tetracycline biosynthesis (ko00253), vancomycin antibiotic synthesis (ko01055), and cytochrome P450 metabolism of xenobiotics (ko00980), which are known to modulate diseases relevant to humans (Table 2).

**Table 2:** Bacteriocin, immunoregulation and related genes in the genomes of TE0907 and TE1809.

		***L. plantarum* TE0907**	***L. plantarum* TE1809**
**Pathway**	**Function**	**Gene numbering**	**Gene numbering**
ko00253	Tetracycline biosynthesis	*PROKKA_00879,* *PROKKA_00880,* *PROKKA_00881,* *PROKKA_00882,* *PROKKA_01391,* *PROKKA_01392,* *PROKKA_01393,* *PROKKA_01395,* *PROKKA_02686*	*PROKKA_00154,* *PROKKA_00155,* *PROKKA_00156,* *PROKKA_00158,* *PROKKA_02402,* *PROKKA_02403,* *PROKKA_02404,* *PROKKA_02405,* *PROKKA_02650*
ko00980	Metabolism of xenobiotics by cytochrome P450	*PROKKA_00641,* *PROKKA_01022,* *PROKKA_01218,* *PROKKA_01406,* *PROKKA_01407,* *PROKKA_01531,* *PROKKA_01923,* *PROKKA_01991,* *PROKKA_02203,* *PROKKA_02225,* *PROKKA_02529,* *PROKKA_02933*	*PROKKA_00169,* *PROKKA_00170,* *PROKKA_00293,* *PROKKA_01082,* *PROKKA_01527,* *PROKKA_01896,* *PROKKA_01918,* *PROKKA_02118,* *PROKKA_02333*
ko01055	Biosynthesis of vancomycin group antibiotics	*PROKKA_00740,* *PROKKA_02089*	*PROKKA_00677,* *PROKKA_02853*
ko05150	*S. aureus* infection	*PROKKA_00024,* *PROKKA_01615,* *PROKKA_02476,* *PROKKA_02477,* *PROKKA_02478,* *PROKKA_02479*	*PROKKA_00377,* *PROKKA_01473,* *PROKKA_01474,* *PROKKA_01475,* *PROKKA_01476,* *PROKKA_02497*
ko00311	Penicillin and cephalosporin biosynthesis	*PROKKA_01222,* *PROKKA_02592*	*PROKKA_02114,* *PROKKA_02736*
ko03320	PPAR signaling pathway	*PROKKA_00149,* *PROKKA_02679*	*PROKKA_00636,* *PROKKA_02657*
ko04622	RIG-I-like receptor signaling pathway	*PROKKA_00376,* *PROKKA_01078*	*PROKKA_02274,* *PROKKA_02925*

**Figure 5:**
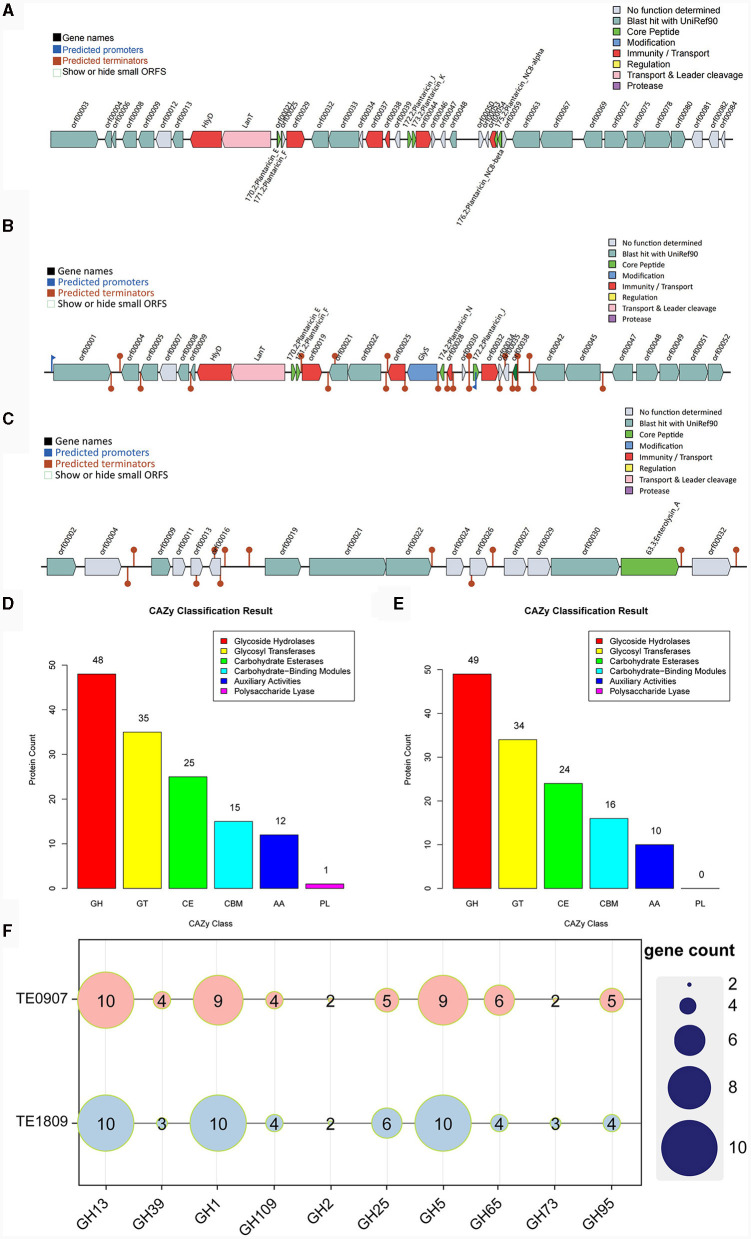
Metabolic system analysis of TE0907 and TE1809. Annotation of bacteriocins in the *L. plantarum* TE0907 and *L. plantarum* TE1809 genome. **(A)** Alignment-based prediction of bacteriocin-related genes encoded within the *L. plantarum* TE0907 genome using the BAGEL4 database; **(B, C)** alignment-based prediction of bacteriocin-related genes encoded within the *L. plantarum* TE1809; **(D, E)** annotation of the *L. plantarum* TE0907 and *L. plantarum* TE1809 genome CAZy database alignment based on HMMER3 (E-value < 1e-5); **(F)** heat map of glycoside hydrolase (GH) distribution in the genomes of *L. plantarum* TE0907 and *L. plantarum* TE1809.

The bacteriocin gene cluster known as Plantaricin was identified in TE0907 and TE1809, respectively. TE0907 exhibited six distinct gene clusters: Plantaricin_E, Plantaricin_F, Plantaricin_J, Plantaricin_K, Plantaricin_NC8-alpha, and Plantaricin_NC8-beta (Figure 5A). Conversely, the *L. plantarum* TE1809 genome unveiled two salient regions: the first encompassing gene clusters Plantaricin_E, Plantaricin_F, Plantaricin_N, and Plantaricin_J, and the second highlighting the Enterolysin_A gene cluster (Figures 5B, C). Multiple open reading frames (ORF) within these bacteriocins underscore a potential correlation between the observed antibacterial characteristics in both strains and the bacteriocins' existence. Employing a genomic lens, we have corroborated the efficacious antibacterial components metabolized by the strain, as initially discerned in our antecedent controlled variable investigations.

The two strains manifest distinct quantities of genes for carbohydrate-active enzymes (CAZymes), with 136 and 133 identified in each, and notably feature a substantial presence of GHs genes, at 48 (35.3%) and 49 (36.1%), respectively, followed by genes for glycosyl transferases (GTs) at 35 (25.7%) and 34 (25.6%), and genes for carbohydrate esterases (CEs) at 25 (18.4%) and 24 (18.0%), respectively, illustrating a diverse enzymatic composition. Furthermore, it is worth noting that only the genome of *L. plantarum* TE0907 has been subjected to gene annotation, explicitly focusing on identifying genes encoding polysaccharide lyases (PLs) (Figures 5D, E). Heatmaps were constructed using the top 10 GHs in terms of abundance from two selected genomes to provide a more intuitive representation of GH distribution. The results indicated that GH1, GH5, and GH13 have a relatively higher prevalence in the genomes of each bacterial strain (Figure 5F). Simultaneously, this revelation underscores the potent metabolic and biosynthetic prowess of the *L. plantarum* strains, thereby elucidating the molecular physiological underpinnings pivotal for their pronounced antibacterial potency.

**Figure 6:**
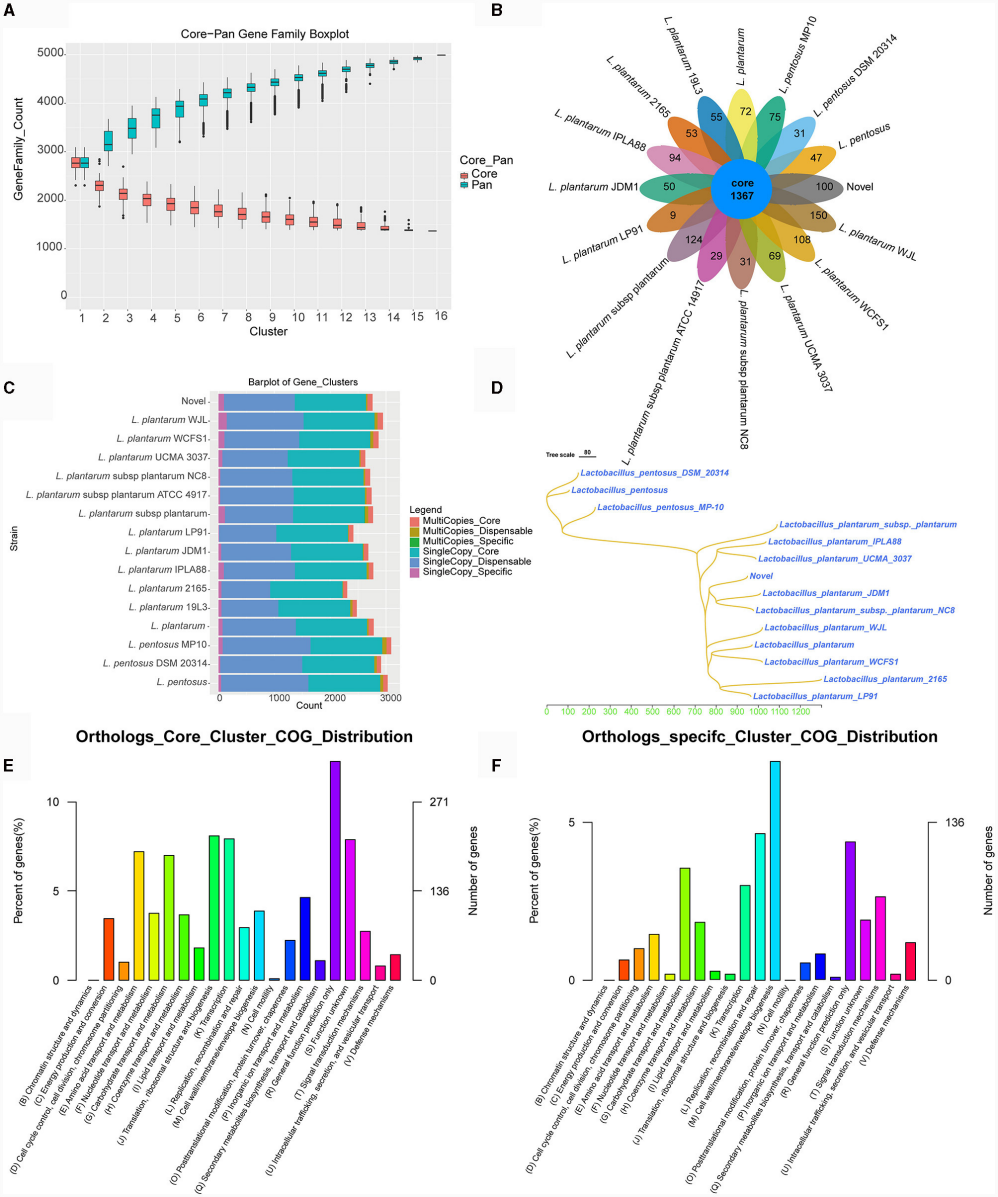
Pan-genome analysis of *L. plantarum* TE0907. **(A)** Box plot of Core-Pan gene dilution (green represents the Pan gene dilution curve, red represents the Core gene dilution curve. The x-axis represents the number of samples selected for each statistical analysis, and the y-axis represents the distribution of the number of samples); **(B)** homologous gene cluster petal diagram (showing the number of shared and unique orthologs clusters); **(C)** bar chart of homologous gene number statistics [single-copy: number of single-copy homologous genes, multiple-copy: number of multiple-copy homologous genes; core: genes that are present in all species; dispensable: genes that are present in some (more than one) species; specific: genes that are present in only one species]; **(D)** a phylogenetic tree diagram constructed using the Neighbor-Joining clustering method based on the pan-genome; **(E, F)** bar chart of COG annotations for shared and unique orthologs clusters.

Melting curve analysis of the genomes of strains TE0907 and TE1809 showcased a coordinated reduction in shared genes and a rise in non-redundant genes (Figure 6A; Supplementary Figure S2A), hinting at an emerging open pan-genome in the *L. plantarum* genomes under investigation. Notably, both strains consistently shared 1,367 and 1,499 genes, respectively, aligning with the gene profiles of all 15 reference *L. plantarum* genomes. Furthermore, with 100 and 153 unique genes in their genomes, they surpassed the gene counts in most other reference strains (100 vs. 9–150; 153 vs. 36–115) (Figure 6B; Supplementary Figure S2B). While 1,367 and 1,499 genes were commonly shared across various *L. plantarum* strains, the distribution of single-copy and multi-copy homologous genes remained consistent: for TE0907, 1267–1285 single-copy (1275 in TE0907) and 72–100 multi-copy (92 in TE0907); for TE1809, 1267–1295 single-copy and 72–100 multi-copy (92 in TE1809) (Figure 6C; Supplementary Figure S2C). These genes chiefly involve cell wall/membrane biogenesis, replication, recombination, repair, and general function prediction (Figures 6E, F; Supplementary Figure S2F). Phylogenetically, amino acid sequences from homologous genes place TE0907 close to plant-associated *L. plantarum* JDM1, while TE1809 aligns closely with *L. plantarum* subsp. *plantarum* ST.III (Figure 6D; Supplementary Figure S1D).

## 4 Discussion

*L. plantarum* strains with superior probiotic properties play a pivotal role in maintaining gut microbial homeostasis, offering targeted treatment against pathogens, and mitigating the overuse of antibiotics (Gurunathan et al., 2023). In this research, through a combination of bacterial genomic sequencing and GC-MS spectrometry analysis, we, for the first time, unveiled the mechanistic actions and associated functional genes of two *L. plantarum* strains derived from *B. gargarizans* in terms of their antimicrobial activity and their capability to modulate the gut microbial composition. This investigation furnishes a more holistic and precise approach to evaluating the efficacy of probiotics, providing novel insights into the field.

The colonization proficiency of gastrointestinal tract (GIT) strains, crucial for their probiotic efficacy, involves not only adherence to mucus and epithelial cells for a competitive advantage, but also the cultivation of substantial interactions with the host, a process essential for probiotic recognition and immune response stimulation, thus significantly magnifying their beneficial effects compared to strains that merely pass through the GIT (Bangotra et al., 2023; Dahiya and Nigam, 2023; Han et al., 2023). In addition, the colonization process by probiotic strains serves as a preventive measure against the attachment of enteric pathogens to the cells of the digestive tract (Son et al., 2023). Extensive research has demonstrated that probiotics exert inhibitory effects on the adhesion of gastrointestinal pathogens, including *S. typhimurium, Clostridium sporogenes*, and *Enterococcus faecalis*, to Caco-2 cells (Bachtarzi et al., 2024; Jain et al., 2023; Lyu et al., 2023). Such adhesion is a prerequisite for the beneficial role of LAB in countering pathogenic bacteria, balancing the intestinal microbiota, and modulating immune responses (Grigoryan et al., 2018; Van Zyl et al., 2020). A key factor in this adhesion process is the probiotic mucin-binding proteins, with the MUB gene playing a central role in adherence to mucin proteins and being a ubiquitous feature in probiotic genomes (Singh et al., 2018). This gene-centric perspective is instrumental in stratifying probiotic strains, emphasizing those with higher expression of MUB gene proteins. Our experiments have highlighted the exceptional adhesion rates of TE0907 and TE1809 to the HT-29 cell line, aligning with existing literature and indicating a fundamental requirement for effective antibacterial action. The distinctive properties of *L. plantarum*, including its strong adhesion to intestinal epithelial cells, ability to persistently colonize, and the antagonistic effects of its metabolites on pathogenic bacteria, solidify its standing as a prototypical probiotic (Dell'Anno et al., 2021; Jeng and Yan, 2022).

Subsequent to meeting the prerequisite of intestinal epithelial adherence, the endeavor progresses toward identifying strains with antimicrobial properties, wherein the assessment of LAB's antibacterial activity typically involves utilizing corresponding pathogenic bacteria as indicator organisms to determine the strains' effectiveness. Our selection was based on the antimicrobial capacity of the isolates against *E. coli, S. aureus*, and *C. jejuni, etc*., which have been linked to numerous foodborne outbreaks (Gray and Killinger, 1966; Lowy, 1998; Kerr and Snelling, 2009; Gomes et al., 2016; Dar et al., 2017), with criteria emphasizing isolates that exhibited a zone of inhibition surpassing that of a control strain, specifically a commercially recognized strain known for producing antimicrobial metabolites (Ahmed et al., 2023). Our goal was to find new potential antimicrobial metabolites-producing strains and ones that could have a more significant potential than cells already reported, when compared to the *L. plantarum* DY1 as reported by Mao et al. (2020), our strains not only exhibit enhanced antibacterial activity but also potentially possess a broader spectrum of microbial inhibition. Probiotic bacteria can render the intestinal environment less conducive to developing pathogens, serving as an additional mechanism for pathogen displacement within the gut. For example, LAB suppress pathogenic bacteria by producing organic acids, antimicrobial peptides, and hydrogen peroxide through metabolism, thereby altering the microbial structure in the intestines and protecting the host's health. Lactic acid in its undissociated state acts as a permeabilizer for the outer cell membrane of Gram-negative bacteria. Once inside the bacterial cytoplasm, it dissociates (Zhang et al., 2023). This dissociation leads to a decrease in intracellular pH level due to the accumulation of ionized forms of organic acids and other antimicrobial substances, ultimately resulting in a bactericidal effect (Jyung et al., 2023). Moreover, short-chain fatty acids (SCFAs), which are vital byproducts of the gut microbiome, have diverse roles. They can modify chemotaxis and phagocytosis, induce reactive oxygen species (ROS), regulate cell division and function, and exert anti-inflammatory, antitumorigenic, and antibacterial effects, besides influencing gut integrity (Tan et al., 2014). Among these SCFAs, acetic acid is notable for being the predominant short-chain fatty acid produced by isolated LAB strains in this study. It is not only a critical metabolic product in the fermentation processes of most bacteria but also a primary substrate for cholesterol synthesis. Additionally, it plays a role in the production of butyric acid (Vidra and Németh, 2018). Interestingly, our findings reveal a striking association (cor ≥ 0.943) between antimicrobial efficacy and acetic acid, a correlation significantly more pronounced than those observed with alternative SCFAs. Consequently, we hypothesize that the strain produces an optimal concentration of acetic acid, which is likely to permeate the cell membranes of *S. haemolyticus* and *Y. enterocolitica*, subsequently lowering the intracellular pH levels. The acidic environment induced by the acetic acid may lead to protein denaturation, disrupting the physiological activities of the bacteria and resulting in widespread cellular dysfunction. This mechanism suggests a targeted antimicrobial strategy that exploits the disruptive potential of acetic acid on pathogenic bacterial cells (Ewadh et al., 2013; Karabiyikli and Sengun, 2017). This association highlight acetic acid's potential central role in antimicrobial activity, warranting further exploration of its mechanistic contributions within microbial ecology and host-microbe dynamics for devising innovative antimicrobial approaches.

LAB-bacteriocins comprise a heterogeneous group of physicochemically diverse ribosomally-synthesized peptides or proteins showing a narrow or broad antimicrobial activity spectrum against Gram-positive bacteria (Choi et al., 2023; Bertuzzi et al., 2024). Bacteriocins, including nisin derived from *L. lactis*, plantaricin generated by *L. plantarum*, and lacticin B obtained from *L. acidophilus*, have antimicrobial activity against food-borne enteropathogens such as *Listeria, Clostridium, Bacillus*, methicillin-resistant *S. aureus*, and vancomycin-resistant *enterococci* (VRE) (Garg et al., 2014; Mathipa and Thantsha, 2017). Bacteriocins can have either a bacteriostatic or a direct bactericidal impact on pathogens, limiting the cells' ability to establish colonization inside the gastrointestinal tract. The antibacterial properties of bacteriocins enable probiotic microorganisms that produce these compounds to acquire a competitive edge within the intricate gastrointestinal milieu (Oftedal, 2023). We conducted the first genomic sequencing analysis of LAB strains from *B. gargarizan*. Furthermore, both strains harbor genes linked to antibiotic biosynthesis, cytochrome P450 metabolism of heterocycles, suggesting their antimicrobial and immune-modulating capacities. Through annotation in the BACEL4 database, we discovered six types of plantaricins were detected in *L. plantarum* TE0907, which have been proven to inhibit pathogenic bacteria such as *S. aureus, Salmonella*, and *E. coli*. Among them, Plantaricin_E and Plantaricin_F form a pair of Class IIb bacteriocins, synergistically exhibiting antimicrobial effects (Wang et al., 2022). The bacteriocin paired with Plantaricin_J is Plantaricin_K. The genome of *L. plantarum* TE1809 contains two potential regions. It is worth noting that these bacteriocins have multiple ORFs, which encode specific immune proteins responsible for bacteriocin secretion, specific proteins involved in modification, and specific proteins involved in transport and leader cleavage. Most ORFs share homology with plnD, plnI, plnH, and plnS, indicating that they may all belong to the pln operon and collectively participate in bacteriocin production (Diep et al., 2009). It is speculated that plantaricin played a significant role in the antimicrobial activity of the selected bacterial strain in this screening. In investigating *L. plantarum*-derived bacteriocins, a complex array of antimicrobial actions has been revealed, primarily focused on undermining the structural and functional integrity of bacterial membranes. Plantaricin FB-2, for example, disrupts the cell membrane, leading to the leakage of vital cytoplasmic substances and an increase in reactive oxygen species (Li et al., 2023). Other plantaricins weaken the proton motive force or create membrane pores, inducing a bactericidal effect on related microorganisms (Wang et al., 2015; Goel and Halami, 2023). Plantaricin A (PlnA) enhances antibiotic effectiveness by increasing bacterial outer membrane permeability and disrupting membrane integrity through interactions with lipopolysaccharides (Meng et al., 2022). These diverse mechanisms highlight the potential of plantaricins as powerful antimicrobial agents, offering a promising approach to combat pathogens and advance microbial therapies.

Upon meticulous examination of metabolic capabilities, the *L. plantarum* strains manifest a pronounced functional enrichment in the realms of carbohydrate metabolism and biosynthesis, with a substantial proportion exceeding 8% of protein-coding genes being intricately associated with carbohydrate transport and metabolic processes. Comparative genomics within the same taxonomic clade elucidates that *L. fermentum* YLF016 and *L. pentosus* ZFM94 are endowed with 6.00 and 8.28% of their genomic repertoire dedicated to such functionalities, respectively (Ye et al., 2020; Zhang et al., 2022). Notwithstanding the species-specific variance in gene abundance, a juxtaposition of the two strains reveals a comprehensive array of genes implicated in carbohydrate and lactic acid metabolism. This discovery further corroborates prior academic investigations into the probiotic mechanisms attributed to the metabolic derivatives of *Lactobacillus* strains, reinforcing their role as the fundamental molecular underpinnings for inhibiting pathogens and bolstering host gastrointestinal wellbeing (Sakaridis et al., 2012).

## 5 Conclusion

We have elucidated, for the first time, the antibacterial efficacy and underlying mechanism of *L. plantarum* derived from *B. gargarizans* utilizing multi-omics approaches, enhancing our comprehension of the diverse array of chemicals and enzymes produced by these microorganisms, as a result, the range of potential uses. The conspicuous antimicrobial attributes manifested in strains TE0907 and TE1809 can largely be ascribed to their salient adhesive prowess and metabolic vigor. Of significance, the resultant antimicrobial dynamism is chiefly propelled by the generation of acetic acid coupled with the production of class II and III bacteriocins.

## Data availability statement

The datasets presented in this study can be found in online repositories. The names of the repository/repositories and accession number(s) can be found in the article/Supplementary material.

## Author contributions

FH: Data curation, Formal analysis, Visualization, Writing – original draft, Writing – review & editing. YZ: Writing – review & editing. YH: Writing – review & editing. YY: Data curation, Writing – review & editing. BY: Writing – review & editing, Funding acquisition, Methodology, Project administration, Resources. XZ: Methodology, Project administration, Resources, Writing – review & editing, Data curation.

## Supplementary material

Supplementary Figure S1 Genome functional annotation of *L. plantarum* TE1809. **(A)** Chromosome Circos plot of the *L. plantarum* TE1809 genome (Annotation as in Figure 4A); **(B–E)** NR, COG, GO, KEGG database annotation of the *L. plantarum* TE1809 genome based on NCBI Blast+ alignment.

Supplementary Figure S2 Pan-genome analysis of *L. plantarum* TE1809. **(A)** Box plot of Core-Pan gene dilution (green represents the Pan gene dilution curve, red represents the Core gene dilution curve. The *x*-axis represents the number of samples selected for each statistical analysis, and the *y*-axis represents the distribution of the number of samples); **(B)** homologous gene cluster petal diagram (showing the number of shared and unique orthologs clusters); **(C)** bar chart of homologous gene number statistics [Single-copy: number of single-copy homologous genes, Multiple-copy: number of multiple-copy homologous genes; Core: genes that are present in all species; Dispensable: genes that are present in some (more than one) species; Specific: genes that are present in only one species]; **(D)** A phylogenetic tree diagram constructed using the Neighbor-Joining clustering method based on the pan-genome; **(E, F)** Bar chart of COG annotations for shared and unique orthologs clusters.

Supplementary material S1 Genomic sequences of bacterial strains.

Supplementary material S2 The sensitivity testing results of TE0907 and TE1809 against seven common antibiotics [sensitive (S), intermediate (I), and resistant (R), with antimicrobial disk concentrations in μg per disk and inhibition zone diameter criteria in millimeters] are based on the standards established by the Clinical and Laboratory Standards Institute (CLSI) of the United States.

Supplementary material S3 Genomic chromosomal circos plot annotation file.
