# Supplementary material for: Unraveling the antimicrobial potential of Lactiplantibacillus plantarum strains TE0907 and TE1809 sourced from Bufo gargarizans: advancing the frontier of probiotic-based therapeutics
Source: Front Microbiol. 2024 Feb 14;15:1347830. doi: 10.3389/fmicb.2024.1347830 (PMC10899456; doi:10.3389/fmicb.2024.1347830)
Supplement: Supplementary material S1 — Genomic sequences of bacterial strains. [file Data_Sheet_1.docx]

**TE0907:** CGGCCGTTGCGGGCGTGCTATACATGCAGTCGAACGAACTCTGGTATTGATTGGTGCTTGCATCATGATTTACATTTGAGTGAGTGGCGAACTGGTGAGTAACACGTGGGAAACCTGCCCAGAAGCGGGGGATAACACCTGGAAACAGATGCTAATACCGCATAACAACTTGGACCGCATGGTCCGAGCTTGAAAGATGGCTTCGGCTATCACTTTTGGATGGTCCCGCGGCGTATTAGCTAGATGGTGGGGTAACGGCTCACCATGGCAATGATACGTAGCCGACCTGAGAGGGTAATCGGCCACATTGGGACTGAGACACGGCCCAAACTCCTACGGGAGGCAGCAGTAGGGAATCTTCCACAATGGACGAAAGTCTGATGGAGCAACGCCGCGTGAGTGAAGAAGGGTTTCGGCTCGTAAAACTCTGTTGTTAAAGAAGAACATATCTGAGAGTAACTGTTCAGGTATTGACGGTATTTAACCAGAAAGCCACGGCTAACTACGTGCCAGCAGCCGCGGTAATACGTAGGTGGCAAGCGTTGTCCGGATTTATTGGGCGTAAAGCGAGCGCAGGCGGTTTTTTAAGTCTGATGTGAAAGCCTTCGGCTCAACCGAAGAAGTGCATCGGAAACTGGGAAACTTGAGTGCAGAAGAGGACAGTGGAACTCCATGTGTAGCGGTGAAATGCGTAGATATATGGAAGAACACCAGTGGCGAAGGCGGCTGTCTGGTCTGTAACTGACGCTGAGGCTCGAAAGTATGGGTAGCAAACAGGATTAGATACCCTGGTAGTCCATACCGTAAACGATGAATGCTAAGTGTTGGAGGGTTTCCGCCCTTCAGTGCTGCAGCTAACGCATTAAGCATTCCGCCTGGGGAGTACGGCCGCAAGGCTGAAACTCAAAGGAATTGACGGGGGCCCGCACAAGCGGGTGGAGCATGTGGGTTTAATTCGAAGCTACGCGAAGAACCCTTACCAGTCTTGACATACTATGCA

**TE1809:** CGCAGTGGGGGAGCTATAATGCAGTCGAACGAACTCTGGTATTGATTGGTGCTTGCATCATGATTTACATTTGAGTGAGTGGCGAACTGGTGAGTAACACGTGGGAAACCTGCCCAGAAGCGGGGGATAACACCTGGAAACAGATGCTAATACCGCATAACAACTTGGACCGCATGGTCCGAGTTTGAAAGATGGCTTCGGCTATCACTTTTGGATGGTCCCGCGGCGTATTAGCTAGATGGTGGGGTAACGGCTCACCATGGCAATGATACGTAGCCGACCTGAGAGGGTAATCGGCCACATTGGGACTGAGACACGGCCCAAACTCCTACGGGAGGCAGCAGTAGGGAATCTTCCACAATGGACGAAAGTCTGATGGAGCAACGCCGCGTGAGTGAAGAAGGGTTTCGGCTCGTAAAACTCTGTTGTTAAAGAAGAACATATCTGAGAGTAACTGTTCAGGTATTGACGGTATTTAACCAGAAAGCCACGGCTAACTACGTGCCAGCAGCCGCGGTAATACGTAGGTGGCAAGCGTTGTCCGGATTTATTGGGCGTAAAGCGAGCGCAGGCGGTTTTTTAAGTCTGATGTGAAAGCCTTCGGCTCAACCGAAGAAGTGCATCGGAAACTGGGAAACTTGAGTGCAGAAGAGGACAGTGGAACTCCATGTGTAGCGGTGAAATGCGTAGATATATGGAAGAACACCAGTGGCGAAGGCGGCTGTCTGGTCTGTAACTGACGCTGAGGCTCGAAAGTATGGGTAGCAAACAGGATTAGATACCCTGGTAGTCCATACCGTAAACGATGAATGCTAAGTGTTGGAGGGTTTCCGCCCTTCAGTGCTGCAGCTAACGCATTAAGCATTCCGCCTGGGGAGTACGGCCGCAAGGCTGAAACTCAAAGGAATTGACGGGGGCCCGCACAAGCGGTGGAGCATGTGGTTTAATTCGAAGCTACGCGAAGAACCTTACCAGGTCTTGACATACTATGCAAATCTAA
